# Aberrant left coronary artery from the pulmonary artery with patent ductus arteriosus - a case report and review of the literature

**DOI:** 10.1186/s13019-024-02803-x

**Published:** 2024-06-05

**Authors:** Ahmad Amer, Hanita Shai, Sagi Assa, Avital Mitler, Alona Raucher Sternfeld

**Affiliations:** 1 Pediatric Cardiology Unit, Wolfson Medical Center, Halohamim 62, Holon, Israel; 2 Faculty of Medicine, Tel-Aviv University, Tel-Aviv, Israel

**Keywords:** ALCAPA, PDA, Congenital heart disease

## Abstract

**Background:**

Aberrant left coronary artery from pulmonary artery (ALCAPA) is a very rare congenital heart defect. Its coexistence with patent ductus arteriosus (PDA) is extremely rare. The high pressures created by the left-to-right shunt in the pulmonary arteries can delay symptoms and create a real challenge in diagnosing ALCAPA. Missing this diagnosis can have severe results, including extensive ischemia and sudden death.

**Case presentation:**

We present a case of an infant born with a large PDA. Initially treated conservatively, however, due to congestive heart failure and lack of weight gain, she underwent surgical ligation of the PDA at the age of four and a half months. Following surgery, she developed pulmonary edema. Echocardiography revealed decreased ventricular function. ECG revealed ST elevations on lateral leads, and serum troponin was significantly increased. The patient underwent cardiac magnetic resonance imaging (MRI), which revealed signs of wall ischemia and decreased function of the left ventricle (LV) with unclear coronary anatomy. Diagnostic catheterization revealed an ALCAPA. She underwent surgical intervention, and the left coronary artery was re-implanted in the aortic sinus. Follow-up revealed slow improvement of cardiac function.

**Discussion and literature review:**

The coexistence of PDA and ALCAPA is a very rare occurrence. We found at least 10 reported cases in the literature. Delayed diagnosis might be detrimental. The prognosis of these patients is variable.

**Conclusion:**

An unusual post-surgical course following PDA repair requires a high index of suspicion and appropriate evaluation for ALCAPA, preferably with angiography.

## Background

ALCAPA, also known as Bland-White-Garland syndrome, is a rare coronary abnormality, constituting 0.3–0.5% of congenital heart defects [1–3]. Left untreated, it can cause mortality in 90% of cases [2, 3]. Survival into adulthood is probably due to collateral development in the coronary circulation, with the risk of sudden death remaining high [4–6].

ALCAPA is, in most cases, an isolated defect, but in rare incidences, it has been described with other anomalies like tetralogy of Fallot, coarctation of the aorta, ventricular septal defects, and PDA (7–8).

ALCAPA is asymptomatic in the fetal period, as the pressures and oxygen saturations are similar in the great arteries. Following birth, the pressure in the pulmonary artery gradually decreases to a point where the coronary perfusion pressure, that is the difference between the pressure in the coronary artery and the LV wall pressure, is not adequate and ischemia ensues. This, in turn, causes dysfunction of the left ventricle, dilation of the left side of the heart, and mitral regurgitation, reflected as clinical heart failure and, in some cases, arrhythmias and sudden death [1, 9].

We report a case of ALCAPA masked by a large PDA in an infant female. Informed consentwas acquired from the patient’s guardian for this report.

## Case presentation

A female infant was born with a large PDA. She developed early congestive heart failure and was initially treated conservatively with diuretics, ACE inhibitors, and high-caloric feeding, and followed up in the pediatric cardiology clinic.

**Fig. 1:**
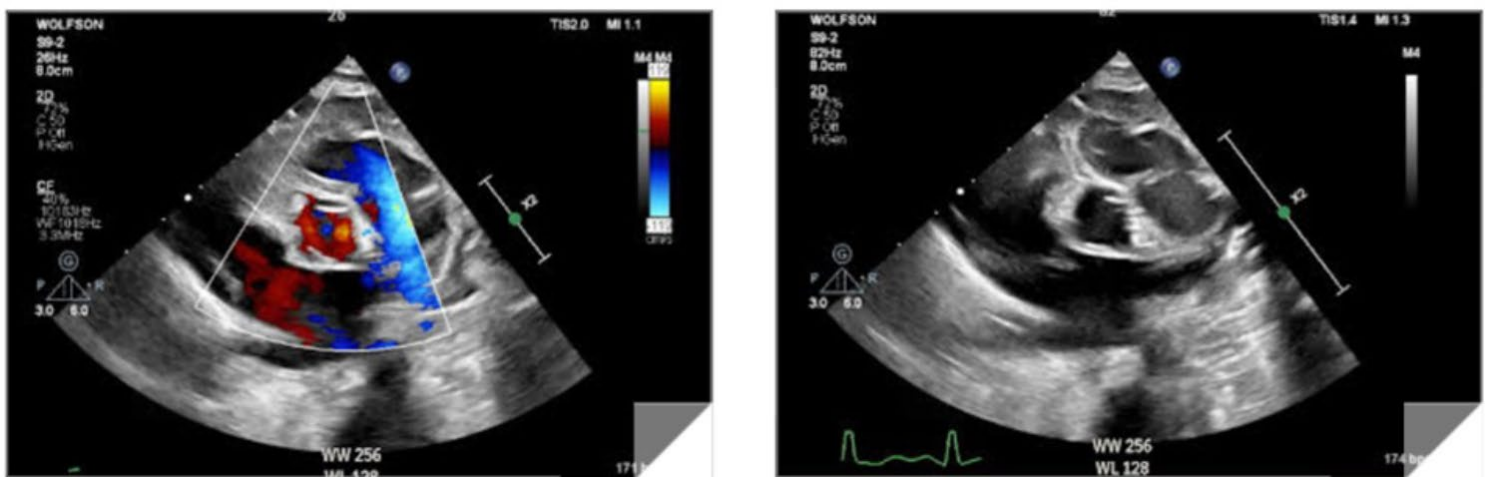
Coronary echocardiographic imaging prior to surgery

Repeat echocardiography showed a PDA 4–5 mm in diameter with a left-to-right shunt across and a low-pressure gradient. The left atrium, left ventricle, and pulmonary arteries were all dilated, as expected. Cardiac function was normal and hyperdynamic in some tests. The coronaries were reported to be normal in origin and course in multiple tests by experienced sonographists and pediatric cardiologists (Fig. 1).

The original management plan was to allow the patient to gain enough weight, making trans-catheter device occlusion of the PDA feasible. However, weekly follow-ups indicated worsening congestive heart failure, reflected by failure to thrive as well as recurrent respiratory infections requiring hospitalization.

Following prolonged hospitalization due to RSV bronchiolitis at the age of 4 months and the persistent lack of weight gain, it was decided to perform surgical ligation of the PDA. At the age of 4.5 months, the PDA was ligated through a lateral thoracic incision with an unremarkable surgical course.

**Fig. 2:**
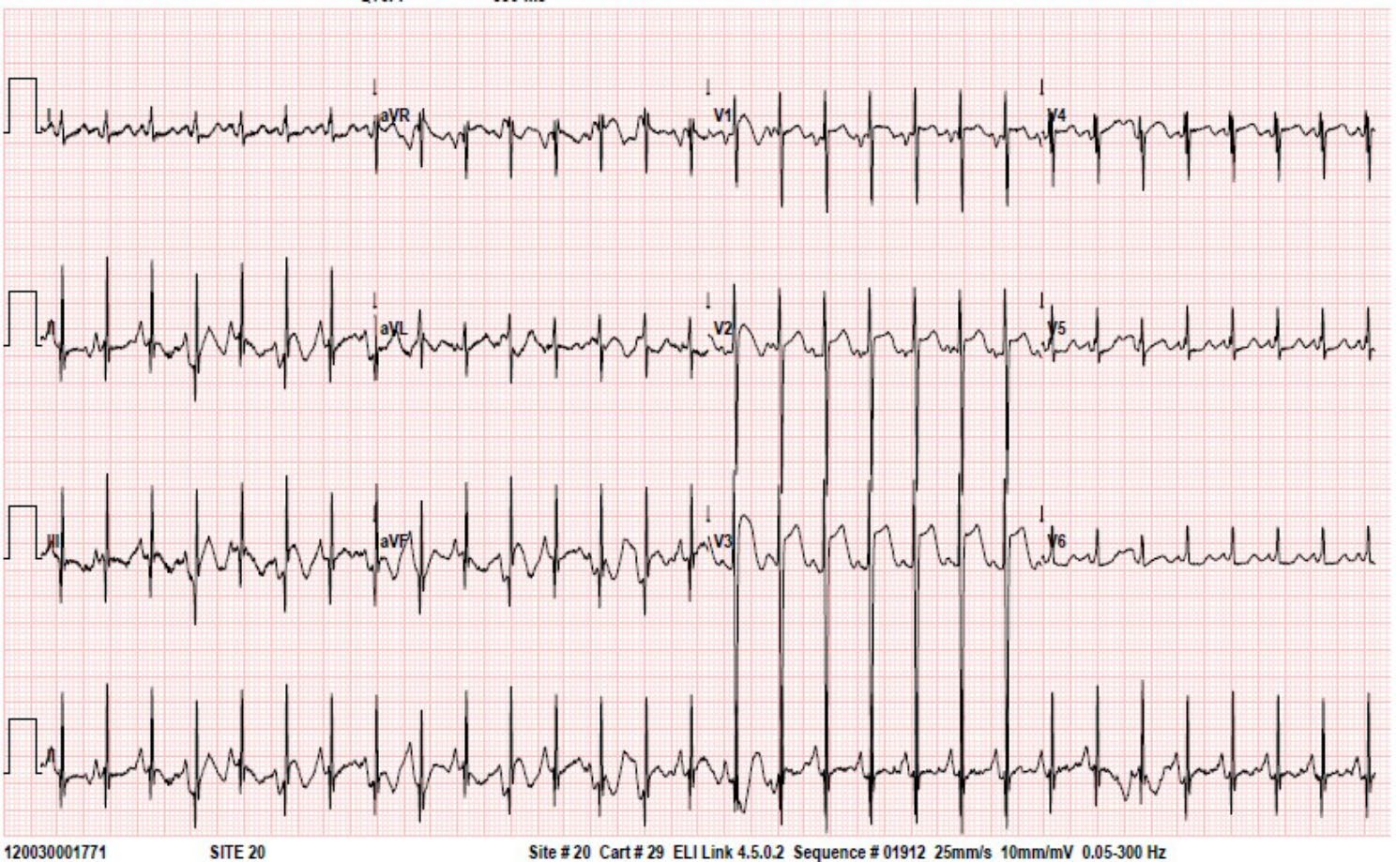
ECG on POD 4revealing ST segment elevations

Following surgery, on post-surgical day (POD) 2, the patient was weaned off mechanical ventilation and catecholamine support. However, dyspnea, tachypnea, and tachycardia persisted. Chest X-ray on POD 3 revealed worsening congestion. Additionally, the ECG which had been normal up to POD 2, began to exhibit pronounced ST elevations on anterior and lateral leads on POD 3 and 4 (Fig. 2). Echocardiography revealed significantly decreased left ventricle function, assessed through “eyeballing” and fractional shortening measurement of 10–15%. Echocardiography did not explain the findings; the coronary anatomy was investigated thoroughly by multiple pediatric cardiologists and reported as normal. Troponin was measured and found to be significantly increased, reaching a maximum of 5330 ng/L.

A differential diagnosis was suggested, including an iatrogenic surgical cause, infectious myocarditis, and coronary anomalies missed in the echocardiography.

**Fig. 3:**
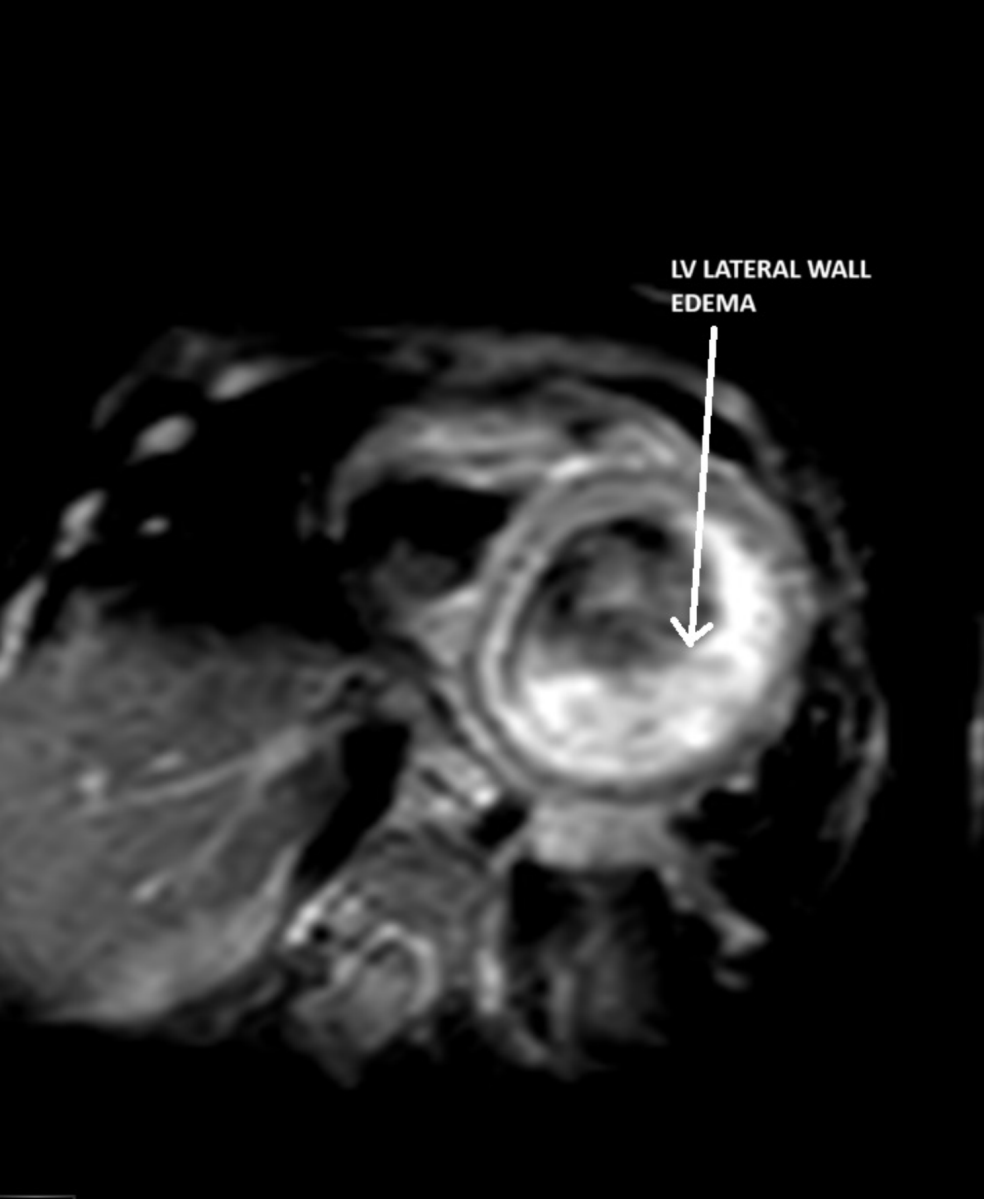
Cardiac MRI scan T2-weighted signal- Lateral LV wall edema

**Fig. 4:**
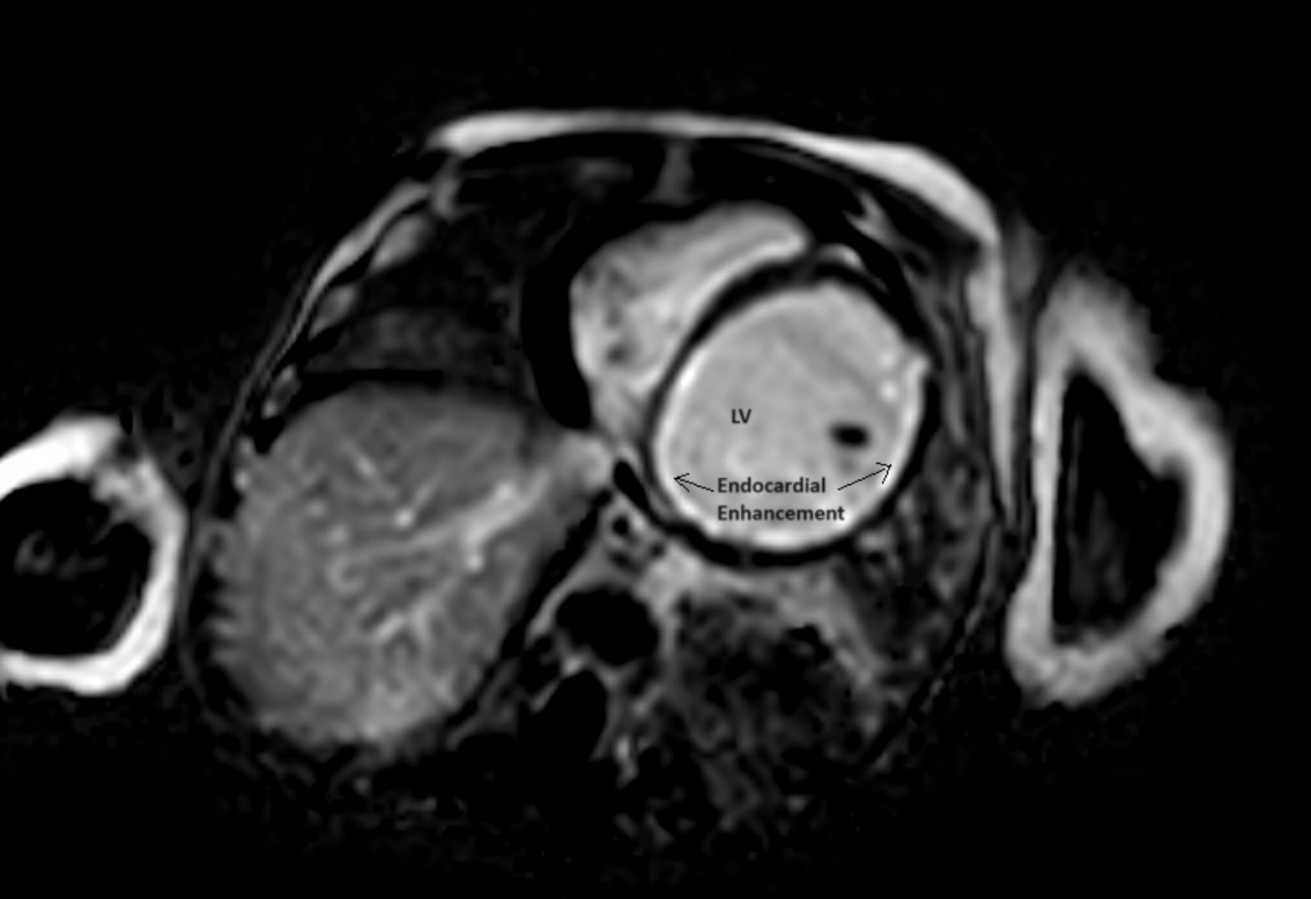
Cardiac MRI scan - Diffuse LV late gadolinium enhancement

Following an extensive interdisciplinary consultation, during which the possibility of conducting further diagnostic tests such as computed tomography (CT) imaging was entertained, it was ultimately determined that pursuing a cardiac magnetic resonance imaging (MRI) examination would offer the most clinically informative approach, particularly in the context of the complex array of potential differential diagnoses under consideration. Subsequently, on POD 6,the patient underwent a cardiac MRI, which revealed thinning of the myocardium and hypokinesia of the lateral wall and septum, increased T2- weighted signal indicating edema, and late gadolinium enhancement of the endocardium (Fig. 3–4). The left ventricle and atrium were dilated, with a left ventricular ejection fraction measured at 20%. The coronary anatomy was not clear.

**Fig. 5:**
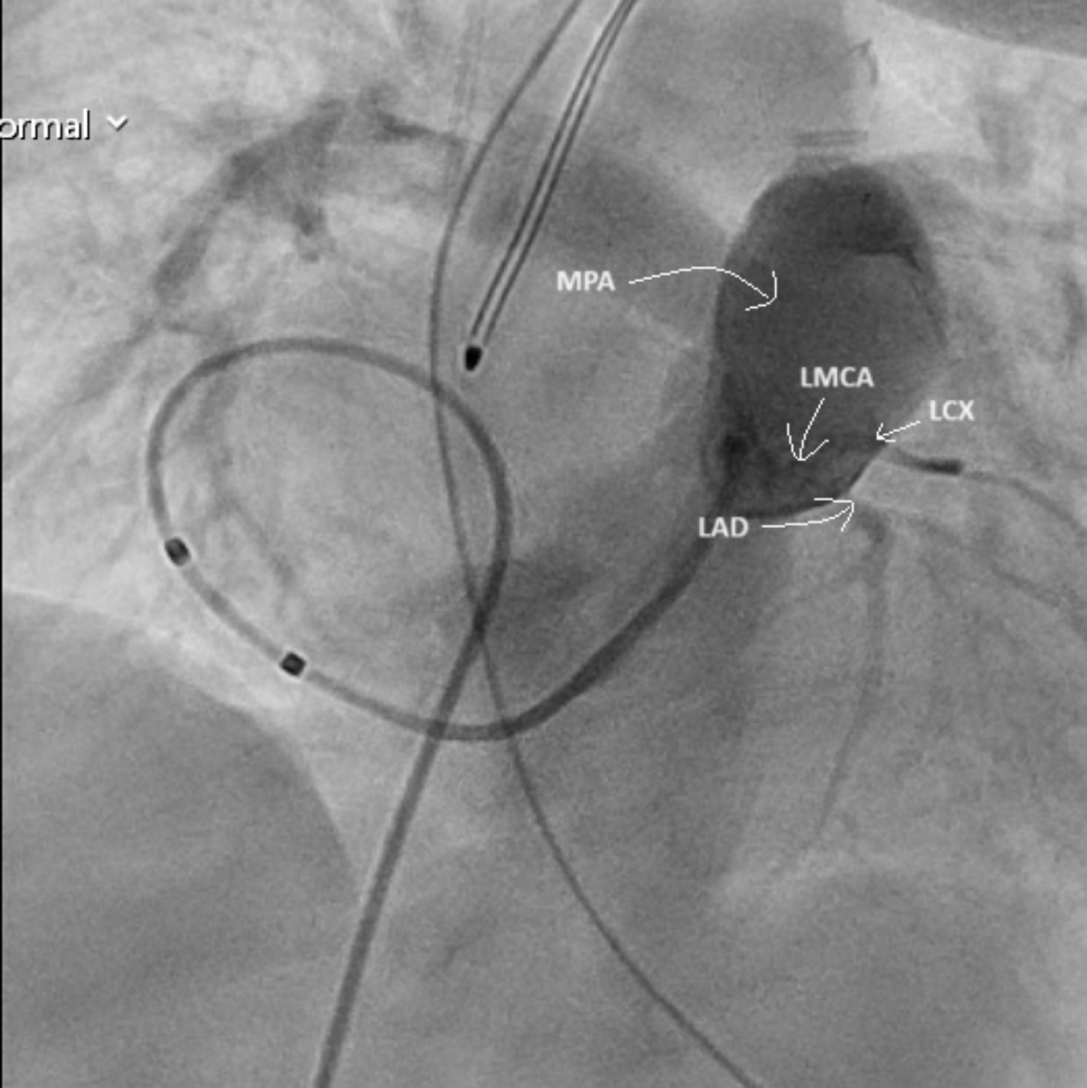
Pulmonary artery angiography – LMCA arising from the pulmonary artery

On POD 7, she underwent pulmonary artery angiography, confirming the diagnosis of ALCAPA (Fig. 5).

She underwent emergent surgical intervention on the same day, during which the left coronary artery was successfully re-implanted in the aortic sinus. The surgery was without complications. She was weaned off cardiopulmonary pump easily and transferred to the pediatric intensive care unit (PICU).

Following surgery, she had a prolonged course due to decreased LV function. Extubation was performed on POD 5, and she was completely weaned off catecholamine support by POD 12. Her PICU length of stay was 25 days, and the total length of stay was 30 days. Prior to discharge, troponin decreased to 358 ng/L, and echocardiography revealed some improvement of cardiac function.

Since her discharge, seven months ago as of the writing of this report, she has been re-hospitalized a total of 7 times. The first re-hospitalization has been just 5 days following her discharge. Most readmissions were due to social factors; however, in two cases, she had respiratory infections with decompensation of her heart failure requiring PICU hospitalization.

Follow-up revealed slow and gradual improvement of cardiac function with wall motion abnormalities and dilated cardiomyopathy. Due to slow improvement, she completed a coronary CT and invasive angiography, both demonstrated patent coronaries arising from the aorta. Her last follow-up was at the age of eleven months, seven months following surgery. She appears well with only mild tachypnea and is gaining weight slowly. Echocardiography revealed dilated LV and left atrium, lateral and septal wall motion abnormalities, and fractional shortening of 23%. The re-implanted left coronary artery appeared patent with normal flow. She continues medical treatment and receives furosemide, captopril, and digoxin, which she tolerates well.

## Discussion and literature review

The combination of ALCAPA and PDA is extremely rare. One paper by Wesselhoeft et al., reviewing 140 cases of ALCAPA, found only one case associated with PDA [9].

The pathophysiology of ALCAPA is usually a gradual decrease of blood supply to the myocardium as the pulmonary pressure decreases following birth [9].

The presence of a sizable arterial duct with a large shunt into the pulmonary artery increases the pressure in the vessel which in turn sustains sufficient perfusion to the left coronary artery, consequently delaying clinical presentation until the duct is ligated [10]. The combination of PDA and ALCAPA is thus gravely dangerous, as the PDA not only complicates the diagnosis of ALCAPA but can also produce catastrophic ischemia when it is repaired (11–10, 12).

We reviewed the literature for all available publications reporting on a combination of PDA and ALCAPA in infants. We searched PubMed for the terms PDA AND ALCAPA and augmented our search with Google Scholar and the regular Google search engine. We chose to omit adult cases, given that the pathophysiology might be different. We found at least 10 reported cases in the literature, which are presented in Table 1.

**Table 1:** Cases of PDA and ALCAPA in literature

Author	Year	Age at presentation	Echocardiography prior to intervention	Clinical presentation after PDA closure	Timing of Diagnosis after PDA closure	Diagnosis modalities	Survival	Follow up
Ortiz et al. [11]	1986	1 year	Large PDA, dilated LA and LV, bicuspid aortic valve, abnormal movement of posterior MV leaflet	CHF, dilated cardiomyopathy	2 years	Angiography	Died	Not applicable
Sreeram et al. [10]	1989	1 month	Large PDA, dilated LA	CHF, decreased LV function, MR	POD 2	TTE	Died	Not applicable
Kiliç et al. [13]	2002	4 months	Large PDA, pulmonary hypertension, dilated RV and moderate TR	Not applicable	Prior to surgery	Angiography	Not reported	Not reported
Bafani et al. [14]	2007	1 month	Large PDA, multiple VSDs, ASD, mild MR	VF, ECG ischemic changes, decreased LV function	Unclear	Angiography	Survived	Not reported
Law et al. [15]	2009	10 days	Large PDA, ASD, dilated LV, moderate TR and mild MR	Decreased LV function, moderate MR, retrograde flow in LMCA	POD 16	TTE	Survived	Normalized LV function
Awashty et al. [16]	2010	4 months	Large PDA, mild MR, hyperechoic PM, ante-grade flow in the LMCA, ALCAPA	Not applicable	Prior to surgery	TTE	Survived	Not reported
Aggarwal et al. [17]	2013	5 month	Moderate PDA, LV dilation, severe MR, hyperechoic PM	Not applicable	Prior to surgery	Angiography	Survived	Normalized LV function
Fedulu et al. [18]	2015	5 months	Large PDA, dilated and hypertrophied LV, Hyperechoic PM, mild-moderate MR	VF, hemodynamic instability	POD 1	TEE	Survived	“Alive and well”
Walker et al. [12]	2016	7 days	Large PDA, mildly depressed LV function, mild MR, hyperechoic PM	Hemodynamic instability, decreased LV function, worsening MR, to and fro flow in the LMCA	POD 0	Angiography	Survived	Almost normalised LV function
Bing et al. [19]	2022	Birth	Large PDA, hypo-plastic aortic arch, VSD, ASD	Failure to wean off CPB	Immediately after repair in the OR	Visual	Died	Not applicable
**Our case**	**2022**	**4 months**	**Large PDA, hyper dynamic function, dilated LV and LA**	**CHF, ECG changes, decreased LV function, high troponin levels**	**POD 7**	**Angiography**	Survived	**Decreased LV function with mild CHF**

LA: left atrium, VSD: ventricular septal defect, ASD: atrial septal defect, MR: mitral regurgitation, PM: papillary muscle, LMCA: left main coronary artery, CHF: congestive heart failure, VF: ventricular fibrillation, ECG: electrocardiography, CPB: cardiopulmonary pump, TTE: trans-thoracic echocardiography, TEE: trans-esophageal echocardiography

Most infants initially present with congestive heart failure (CHF) in the first months of life. All cases, including ours, report normal LV function in the initial presentation; some have mitral regurgitation (MR), which, along with the CHF, is attributed to the large PDA. A specific finding of hyperechoic papillary muscle was reported in four of the ten cases [12, 16–18].

ALCAPA was recognized in three cases prior to surgery. In two cases, it was recognized incidentally during diagnostic catheterization [13, 17]. In the third case, it was diagnosed by transthoracic echocardiography (TTE) [16].

The clinical presentation following PDA closure is dramatic in all cases. Most develop immediate hemodynamic shock or ventricular arrhythmia [12, 14, 18, 19], and some develop CHF with severely decreased LV function (11–10, 15). The diagnosis of ALCAPA following PDA repair might be delayed, especially in cases with a non-catastrophic presentation. In one case, the diagnosis was delayed for 2 years [11]; however, this was an out-of-country case where the local staff might have had limited resources. In another case, the infant improved clinically following PDA closure, and the diagnosis was only made 16 days following the surgery during a routine TTE [15]. In our case, the patient didn’t present dramatically following the PDA closure. As mentioned, she was weaned off catecholamine support and invasive ventilation and developed pulmonary edema on POD 3 and 4. This relatively mild course might have contributed to the delay in the diagnosis.

Angiography remains the gold standard for ALCAPA diagnosis; this is also reflected in the cases described in this paper. TTE can be very effective in finding this diagnosis, but the combination with PDA might limit its utility. This observation may be attributed to the substantial jet generated by the PDA into the pulmonary artery, along with the normal flow direction in the aberrant coronary artery [8]. In our case, apart from TTE, the first modality chosen was cardiac MRI. This was to rule out other considered diagnoses, specifically myocarditis. MRI use is not common in this context. In our case, though it didn’t directly reveal the ALCAPA, it strongly suggested it, and the diagnosis was finalized via angiography as mentioned before.

The prognosis of these patients is variable. Three patients died: one was diagnosed with a significant delay of two years and didn’t survive the surgery [11], another, diagnosed on POD 2 after duct ligation, also didn’t survive the re-implantation procedure [10]. Both of these cases are from the 80s; surgical and anesthesia techniques and expertise have evolved since. The third case of mortality described an infant with severe comorbidity, a hypoplastic aortic arch, who was diagnosed in the operating room following weaning off bypass; he also didn’t survive the surgery [19].

For surviving patients, prognosis appears to be good, although it was only described in four of the seven living cases [12, 15, 17, 18]. The long-term prognosis of patients with isolated ALCAPA is reported to be good [20, 21]. A large series describing more than 100 patients four years after corrective surgery reported very low mortality and normalization of the LV function in almost all patients [21].

In our case, seven months after surgical repair, the patient unfortunately continues to have a dilated LV with decreased fractional shortening, however, she is gaining weight gradually and managed mostly in the ambulatory setting.

## Conclusion

The combination of ALCAPA and PDA carries a special risk. High awareness must be maintained, and any clues for the diagnosis have to be fully pursued prior to PDA repair. An unusual post-surgical course following PDA repair requires a high index of suspicion and immediate appropriate evaluation for ALCAPA, preferably with angiography.

## Data Availability

No datasets were generated or analysed during the current study.

## Glossary of Abbreviations

ALCAPA: Aberrant Left Coronary Artery from Pulmonary Artery
CHD: Congenital Heart Disease
VSD: Ventricular Septal Defect
PDA: Patent Ductus Arteriosus
LV: Left Ventricle
TTE: Transthoracic Echocardiography
PICU: Pediatric Intensive Care Unit
POD: Post-Operative Day
CHF: Congestive Heart Failure
MR: Mitral Regurgitation
LMCA: Left Main Coronary Artery
